# Review of Waterproof Breathable Membranes: Preparation, Performance and Applications in the Textile Field

**DOI:** 10.3390/ma16155339

**Published:** 2023-07-29

**Authors:** Yawen Chang, Fujuan Liu

**Affiliations:** National Engineering Laboratory for Modern Silk, College of Textile and Clothing Engineering, Soochow University, 199 Ren-Ai Road, Suzhou 215123, China; 20225215004@stu.suda.edu.cn

**Keywords:** waterproof breathable membranes, electrospinning, textiles, protective clothing

## Abstract

Waterproof breathable membranes (WBMs) characterized by a specific internal structure, allowing air and water vapor to be transferred from one side to the other while preventing liquid water penetration, have attracted much attention from researchers. WBMs combine lamination and other technologies with textile materials to form waterproof breathable fabrics, which play a key role in outdoor sports clothing, medical clothing, military clothing, etc. Herein, a systematic overview of the recent progress of WBMs is provided, including the principles of waterproofness and breathability, common preparation methods and the applications of WBMs. Discussion starts with the waterproof and breathable mechanisms of two different membranes: hydrophilic non-porous membranes and hydrophobic microporous membranes. Then evaluation criteria and common preparation methods for WBMs are presented. In addition, treatment processes that promote water vapor transmission and prominent applications in the textile field are comprehensively analyzed. Finally, the challenges and future perspectives of WBMs are also explored.

## 1. Introduction

**Figure 1:**
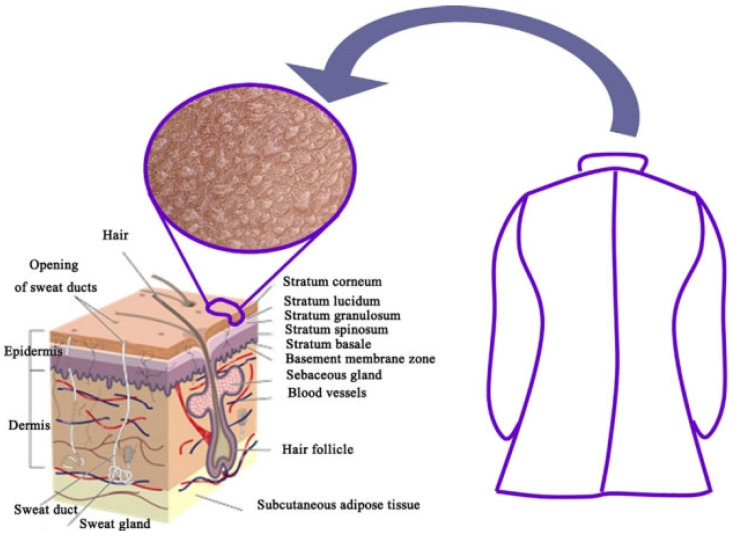
Representative picture of the skin and wearable clothes [3].

Skin is a type of human tissue that comes into direct contact with the external environment, with functions such as protection, respiration, secretion and the perception of external stimuli [1]. Skin allows moist vapor to effectively propagate from the inside to the outer atmosphere, while protecting the human body against the penetration of liquid water such as rainwater and snow. This special property is defined as “breathability”, which is of great significance in regulating the function of organisms. Skin has the ability to regulate body temperature to a certain extent [2], but in extreme environments such as high temperature and extreme cold, the comfort and safety of the human body cannot be guaranteed. Therefore, waterproof breathable materials have been developed to ensure the normal operation of skin. Figure 1 shows the relationship between skin and fabric [3]. Natural fibers were the earliest waterproof breathable materials, but barely meet people’s needs because of their small variety, strong hydrophilicity and poor adhesion to the skin. Compared with natural fibers, synthetic fibers occupy the main market of high-quality functional clothing due to their high strength and no deformation [4]. With the development of science and technology, scientists developed the first waterproof breathable membranes (WBMs) in the 1970s and combined them with textile materials by laminating composite methods, while ensuring the waterproof breathable property and strength of the fabric.

At present, commercial WBMs are generally divided into two types, namely hydrophilic non-porous membranes and hydrophobic microporous membranes. There is no pore structure on the surface and inside of hydrophilic membranes, and liquid water cannot penetrate them. Hydrophobic microporous membranes, more widely used than hydrophilic non-porous membranes, have an appropriate pore size and high porosity. These membranes can distinguish between water droplets (diameter > 100 μm) and small gas molecules (diameter < 1 nm), so as to allow water vapor to transfer from the body to the outside and prevent water penetration [5], thus forming a “microclimate” between the skin and fabric. WBMs provide protection and wearing comfort for the human body, and are widely applied in ski suits, nautical suits [6], military clothing, police jackets [7], etc.

By allowing water vapor to pass through and limiting the entry of liquid water, WBMs not only can be used as a waterproof breathable layer for clothing to ensure human comfort, but have also had significant development in fields such as construction materials [8], batteries [9], water treatment [10] and electronic equipment [11]. For example, in contrast to relatively expensive proton-exchange membranes, WBMs introduce oxygen and remove water vapor on the cathode side of membrane fuel cells (MFCs) [12], which are a kind of gas diffusion layer material with easy preparation, low cost and stable cathode structure. In addition, with the development of science and technology, mechanical and electronic products have begun to use a large number of WBMs to protect internal electronic components, as they can block liquid, dust, etc., from entering the interior [13]. More importantly, compared with completely sealing electronic devices to achieve waterproofing, the breathability of WBMs allows them to remove toxic gases generated by batteries, preventing internal leakage and affecting use.

Moreover, as the demand for fresh water increases, methods of water treatment are constantly developing. Among them, reverse osmosis (RO), forward osmosis (FO) and membrane distillation (MD) are common desalination methods. RO achieves seawater desalination by driving water molecules and ions through a hydrophilic membrane under high pressure [14], and its water transport mechanism is explained by the solution–diffusion model. But recently, Elimelech’s team [15] proposed that the migration of water and solvent in RO can be predicted by a solution–friction model, in which water molecules travel in clusters through tiny transient holes within the polymer that exert friction on them as they pass through. As a permeation-driven membrane process, FO has the characteristics of low energy consumption, good separation effect and simple operation. Al-Furaiji et al. [16] prepared a thin-film composite (TFC) membrane with polyacrylonitrile (PAN) as the supporting layer. PAN-TFC membrane has excellent porosity, water flux and mechanical properties that are no less than those of the RO membrane. MD technology is a combination of membrane separation and thermal distillation [17], which utilizes the temperature difference on both sides of the membrane to generate a vapor pressure difference [18], enabling WBMs to separate liquid feed from a penetrant.

Common methods of preparation and processing of WBMs mainly include melt extrusion, biaxial stretching and electrospinning. The melt extrusion method has strong adaptability and uniform coating, but most processed membranes have poor water vapor permeability. Microporous membranes can be manufactured by biaxial stretching, but the preparation process is complicated and pore size adjustment is difficult. Currently, the electrospinning technique is considered to be the most effective method for fabricating WBMs. By adjusting the process parameters, electrospinning membranes with suitable fiber morphology and pore structure can be produced. In order to obtain WBMs with better comprehensive performance, two modification methods can be applied: doping modification and post-treatment. In the past few years, WBMs have developed rapidly in the textile industry, showing promising application prospects in fields such as outdoor clothing, protective clothing, wound dressing and smart clothing.

In this review, we focus on analyzing the waterproof and breathable mechanisms of two types of WBMs: hydrophilic non-porous membranes and hydrophobic microporous membranes. The advantages and disadvantages of three common WBM preparation methods are compared, and doping modification and post-treatment methods are introduced from the perspective of optimizing waterproof and breathable properties. Then, the latest progress and application of WBMs in the textile field are summarized in detail, including daily outdoor sportswear, special protective clothing, wound dressings and bionic textiles. In addition, the future development trends of WBMs are discussed in the hope that this review can promote a deeper understanding of WBMs and provide more inspiration for its applications in the textile field.

## 2. Mechanisms of Waterproofness and Breathability

WBMs can effectively prevent external water from entering the inside and allow water vapor to transfer from the body to the outside, and are mainly categorized into hydrophilic non-porous membranes and hydrophobic microporous membranes. The different waterproofness and water vapor permeability mechanisms of these two membranes are analyzed. Hydrophilic non-porous membranes prepared by melt extrusion are a kind of continuous non-porous membrane material processed by hydrophilic polymers, such as polyurethane (PU) [19], polyacrylonitrile (PAN) [20,21], cellulose [22,23], etc. Hydrophobic microporous membranes, which apply hydrophobic polymer as the basic raw material and are fabricated by phase separation, biaxial stretching or electrospinning, have a large number of small, connected micrometer-level pores. The polymers used in the process usually involve polytetrafluoroethylene (PTFE) [24], polystyrene (PS) [25], poly(vinylidene fluoride) (PVDF) [26], polyimide (PI) [27], polypropylene (PP) [28], etc. In addition, research into WBMs mainly focuses on hydrophobic microporous membranes.

### 2.1. Hydrophilic Non-Porous Membranes

**Figure 2:**
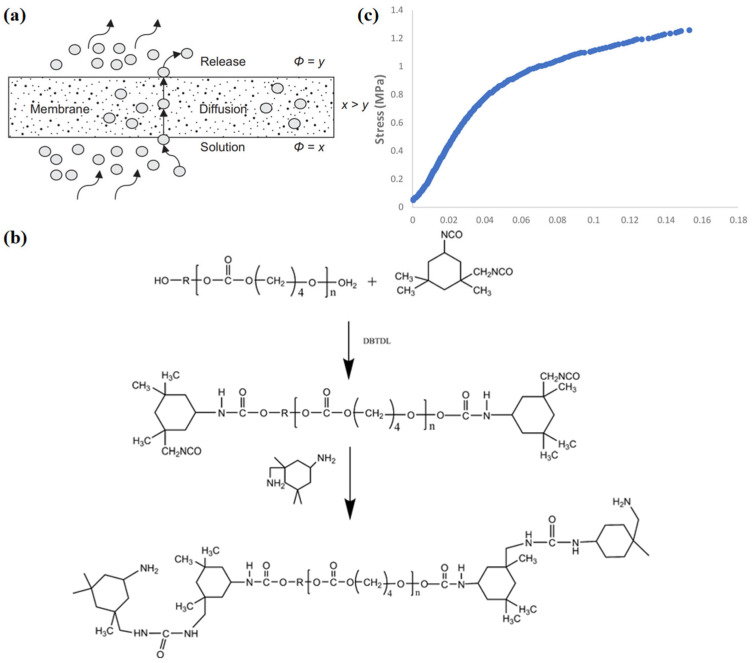
(**a**) Schematic diagram of water molecular transport in a hydrophilic non-porous membrane [32]; (**b**) chemical structures and compositions of polyurethane material [34]; and (**c**) stress and strain relationship of PAN membrane [16].

Achieving waterproof and breathable effects under specific circumstances, hydrophilic non-porous membranes, with no pore structure inside, make liquid water completely unable to penetrate. Because the non-porous structure does not easily absorb dust and other foreign bodies, there is no hole blockage and it can be wet for a long time. Hydrophilic non-porous membranes move water vapor through chemical diffusion. Under a certain temperature and humidity gradient, water molecules are adsorbed on the side with high humidity and transferred to the side with low humidity through hydrophilic groups on the polymer chains for desorption. The diffusion of water vapor is realized by hydrogen bonding of hydrophilic functional groups [29], while the hydrophilic segments (e.g., oxyethylene groups) or side groups (e.g., –OH, –COOH and –NH_2_) [30] are responsible for transfer of water vapor (WV). Fluctuations in polymer chains can accelerate the propagation of water vapor, thereby promoting higher permeability [31]. Due to the higher heat and humidity between the human body and clothing system, a pressure difference is created, forcing heat and moisture to flow outward. Figure 2a [32] illustrates the waterproofness and water vapor permeation mechanisms of a hydrophilic non-porous membrane. Water resistance comes from the continuity and high membrane tension of the hydrophilic non-porous membranes, in which hydrophobic segments inside the membranes play a role in resisting the penetration of external liquid water.

However, hydrophilic groups can capture fewer water molecules and cannot conduct them quickly. Hence, in order to achieve good water vapor permeability, the fabric needs to be close to the skin and in direct contact with sweat, which greatly reduces the comfort of the fabric and limits the application of hydrophilic non-porous membranes [33]. At the same time, after long-term contact with water, the membranes will be thoroughly infiltrated and cause serious deformation, which will affect the water resistance of the membranes as well as the smoothness and beauty of the clothing.

Polyurethane (PU) is a typical thermoplastic elastomer applied in hydrophilic non-porous membranes [35], which has outstanding characteristics such as high mechanical strength, thermal stability, chemical resistance, wear resistance and simple machining. However, high surface energy (40 mJ/m^2^) [36], poor hydrophobicity and low water vapor permeability of PU provide a poor experience for wearers, thus limiting its application to a certain extent. Figure 2b [34] illustrates the process principle diagram of PU synthesis. Isofluorone diisocyanate (IPDI) is dripped into polycarbonate diol (PCDL) and mixed with dilaurate (DBTDL) catalyst. Then, isophorone diamine (IPDA) is used to extend the chain to synthesize PU.

Polyacrylonitrile (PAN) with a C≡N group possesses good thermal stability, electrochemical stability and relatively high hydrophilicity [21], which makes it an important material for the manufacture of hydrophilic non-porous membranes. However, PAN has poor mechanical properties (as shown in Figure 2c [16]) and needs to be combined with other materials to overcome this defect. Cellulose, as the most abundant polysaccharide, is a kind of renewable material with hydrophilicity, biocompatibility and biodegradability. Synthetic cellulose, such as cellulose acetate and cellulose nitrate, is a candidate material for producing hydrophilic nonporous membranes [23].

### 2.2. Hydrophobic Microporous Membranes

**Figure 3:**
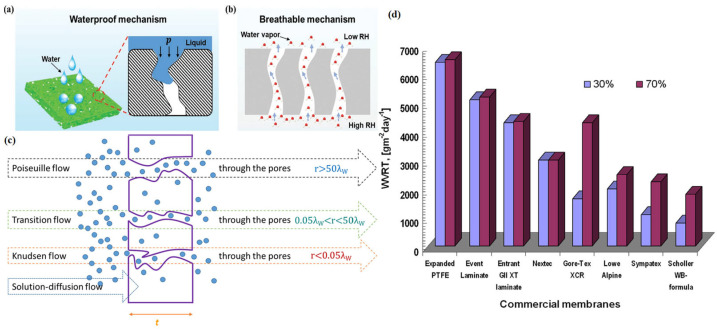
(**a**) Waterproof mechanism; (**b**) breathable mechanism of hydrophobic porous membranes [39]; (**c**) four different mechanisms of water vapor transport through WBMs [45]; and (**d**) comparison of WVRT through commercial membranes in two different relative humidity conditions (RH = 30% and 70%) [3].

Hydrophobic microporous membranes are a kind of WBM based on hydrophobic polymers, while biaxial stretching and electrospinning are the main preparation methods [37]. The low surface energy groups (such as –CF_2_, –CF_3_ and –CH_3_) of hydrophobic polymers provide low surface energy (such as PTFE [17] with a surface energy of only 19.1 × 10^3^ N/m^2^), making their surface hydrophobic [38]. The interior of the membranes is an irregular network/sponge-like structure with a large number of small, connected micron-scale pores [3], which can provide channels for water vapor transfer and prevent external water molecules from entering [39]. Figure 3a–c describes the waterproofness and water vapor permeation mechanisms of hydrophobic microporous membranes. In use, hydrophobic microporous membranes do not need direct contact with the sweat generated by the human body, so membranes will not adhere to the human skin, effectively ensuring the comfort of the human body. However, the pore structure inside the microporous membrane is prone to pollution and blockage, leading to a significant reduction in the water vapor permeability of clothing. It is usually necessary to use a multi-layer composite method of separating it from dust, etc., to guarantee long-term waterproofness and water vapor permeability. Due to their high comprehensive performance, hydrophobic microporous membranes have been widely used in various fields such as protective clothing, medical care [40] and packaging [41].

The original hydrophobic microporous membrane is made of polytetrafluoroethylene (PTFE) resin [18] by unidirectional/biaxial mechanical stretching, which has the commercial name Goretex™. Figure 3d [3] compares the water vapor permeability of common commercial WBMs in two different relative humidity conditions (RH = 30% and 70%). The WVRT of the PTFE membrane was higher than that of other WBMs in both humidity conditions, and the WVRTs of the latter four membranes in a wet environment were significantly higher than those in a dry environment. Due to the existence of fluorine atoms in the PTFE molecular chain [42], the surface tension of the polymer is low, making it an excellent waterproof material. In addition, the presence of fluorocarbon chains makes PTFE non-degradable and polluting [43], which is not environmentally friendly.

As a common non-polar thermoplastic polymer, polystyrene (PS) is polymerized from styrene as a monomer [25]. Owing to its long carbon–hydrogen chain and low surface energy, PS can be used as a hydrophobic material for the manufacture of WBMs, but unfortunately, its poor barrier ability to water vapor restricts its development. Poly(vinylidene fluoride) (PVDF) is widely used in waterproof materials because of its thermal stability, excellent chemical resistance and good waterproof ability [44]. However, due to the inherent low surface energy of PVDF, it easily absorbs organic matter and causes pollution during the application process. Therefore, hydrophilic modification is generally carried out to improve the anti-fouling performance of PVDF membranes.

## 3. Evaluation Criteria for WBMs

The water resistance and water vapor permeability of WBMs are typically characterized by three parameters, namely hydrostatic pressure, water contact angle (WCA) and water vapor transfer rate (WVTR).

### 3.1. Evaluation Criteria for Water Resistance

#### 3.1.1. Hydrostatic Pressure

Hydrostatic pressure is an omnidirectional force evenly applied to all parts of the surface, which is the force required for an object to resist water penetration. It is an important index for observing the water resistance of materials. Increasing hydrostatic pressure will reduce the volume of the object under stress but will not change its shape. Thus, the greater hydrostatic pressure the membrane can withstand, the better the water resistance or leakage resistance.

In theory, the hydrostatic pressure Δ*P* of a membrane can be calculated according to the Young–Laplace equation [46]:

(1) $$\Delta P=\frac{{4\gamma }_{LG}{\cos\theta }_{\mathrm{adv}}}{d_{\max}}$$

where *γ_LG_* is the surface tension of water, *d*_max_ is the maximum pore diameter of the WBM, and *θ*_adv_ is the front influent contact angle of the capillary wall, which is related to the hydrophobicity of the material.

#### 3.1.2. Water Contact Angle (WCA)

Wetting phenomena depend on the ability of a liquid to spread on the surface, which also plays a key role in membrane waterproofness. The degree of wetting (*W*) is expressed as a function of surface energies [47]:

*W* = γ*_SV_* − (γ*_SL_ +* γ*_LV_*) (2)

where *γ_SV_* is the surface free tension between solid and vapor phase, *γ_SL_* is the surface free tension between solid and liquid phase, and *γ_LV_* is the surface free tension between liquid and vapor phase. Positive values of wetting (*W* > 0) mean high hydrophilicity, whilst negative values (*W* < 0) are typical of a hydrophobic surface.

Based on the Young–Laplace equation, it is obvious that the waterproof performance of porous membranes not only depends on *d*_max_ but is also related to the wettability (*θ*_adv_).

**Figure 4:**
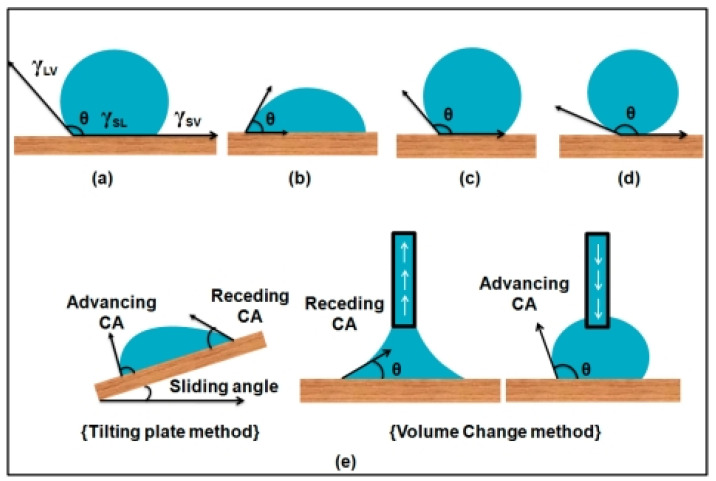
Schematic definition of hydrophilicity, hydrophobicity and super-hydrophobicity: (**a**) interfacial tensions of all three phases that co-exist and static contact angle; (**b**) hydrophilic surface contact angle (*θ*) < 90°; (**c**) hydrophobic surface contact angle(*θ*) > 90°; (**d**) super-hydrophobic surface contact angle(*θ*) > 150°; and (**e**) dynamic contact angles for measurement of contact angle hysteresis [47].

The WCA is defined by the contact area between solid and water, as exhibited in Figure 4 [48]. Interfacial energies play a major role in determining the contact angle between liquid droplets and solid surfaces. The formula for measuring the water contact angle (WCA) is as follows [47]:

(3) $${\cos\theta }_{\mathrm{adv}}=\frac{\gamma_{SV}-{\;\gamma }_{SL}}{\gamma_{LV}}$$

According to the Young–Laplace equation, the wettability of liquid droplets on a solid surface can be quantified numerically—by the WCA. For smooth surfaces, the WCA increases when *γ_SV_* decreases or *γ_LV_* increases. For WCAs less than 90°, a wet surface is more energy favorable than a dry solid surface and is therefore considered hydrophilic. When the WCA is greater than 90°, the surface energy of a dry solid can be more stable, so it is known as hydrophobic. If the WCA is greater than 150°, the surface is identified as super-hydrophobic.

### 3.2. Evaluation Criteria for Water Vapor Permeability

Another undeniable characteristic of WBMs is water vapor permeability, generally quantified by water vapor transfer rate (WVTR). Under steady-state conditions, the flux obeys Fick’s first law and is related to the permeability(*J*) by the following equation [49]:

(4) $$J=P\frac{\left(p_{h}-p_{l}\right)}{l_{m}}$$

where *l_m_* is the membrane thickness, *p_h_* is the upstream pressure (high pressure) and *p_l_* is the downstream pressure (low pressure).

Water vapor transport in porous membranes is driven by vapor concentration gradients that allow water vapor to flow freely through membrane channels from high relative humidity (RH) to low relative humidity [11]. In a porous hydrophobic membrane, water vapor transport is mainly achieved through pores. The higher the water vapor transmission rate (WVTR), the better the water vapor permeability of the membrane. The WVTR can be calculated by the following formula [50]:

(5) $$\mathrm{WVTR}=\frac{\Delta m}{S\;\times \;t}\;\times \;24$$

where Δ*m* is the mass variation of test cups during testing, *S* is the testing area and *t* is the testing time.

## 4. Preparation Methods of WBMs

Depending on the material and structure, waterproof breathable membranes can be fabricated in different ways. There are three main manufacturing methods of WBMs: melt extrusion, biaxial stretching and electrospinning.

### 4.1. Melt Extrusion

**Figure 5:**
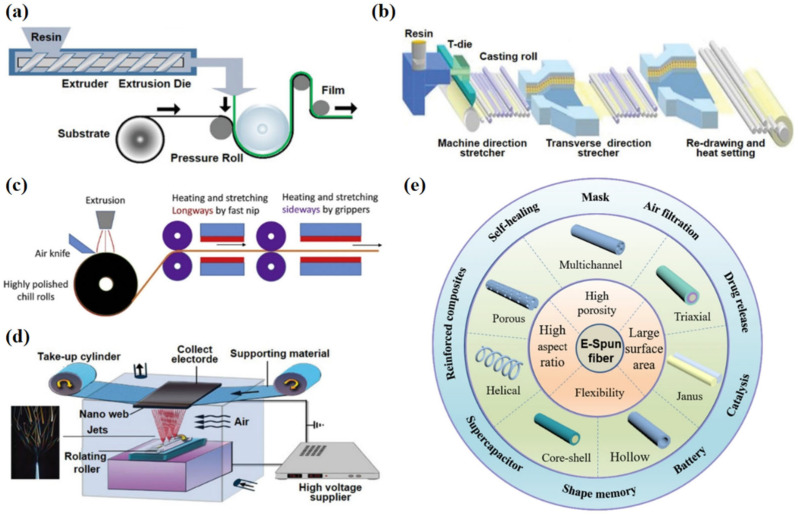
(**a**) Technical diagram of melt extrusion [54]; (**b**,**c**) technical diagram of biaxial stretching method [45,54]; (**d**) technical diagram of electrospinning [54]; and (**e**) the characteristics, structures and applications of electrospinning fibers with different structural designs [56].

Melt extrusion can achieve an effect of melting thermoplastic polymer materials into a membrane through heating and extrusion stretching [51]. It has advantages of low cost, high efficiency, strong material adaptability, good processing continuity, uniform coating and solvent-free application [52], and it is the most widely used processing molding method in the field of polymer processing. Melt extrusion can accurately control the thickness of a membrane through the extrusion speed of the molten polymer and can adjust the orientation direction and crystallinity of the polymer molecular chain in the membrane by changing the ratio of the stretching rate between the warp and the zonal after extrusion. Therefore, the structure and properties of the membrane can be effectively regulated [53]. Figure 5a [54] demonstrates the preparation process of melt extrusion. The water vapor permeability of the hydrophilic non-porous membranes is not limited by the physical structure of the membrane materials, but depends on the hydrophilic groups in the molecular structure of the polymer raw materials used, so melt extrusion is the most suitable way for the production of hydrophilic non-porous membranes [55]. However, there are some limitations to melt extrusion, such as the need for coating weight to reach certain limits, polymer melt instability, coating speed restrictions, etc.

### 4.2. Biaxial Stretching

Biaxial stretching, in which a suitable polymer is extruded on a casting drum to form a membrane that is stretched bidirectionally under optimal conditions [57], is a common method for the manufacture of non-porous polymer membranes and PTFE porous membranes. Polymer precipitation can be induced by solvent exchange, thermal gradient or steam diffusion [58]. Figure 5b,c [45,54] depicts the processing flow of the biaxial stretching method. WBMs manufactured by this method have a large number of “island” structures inside [59], and connected microporous structures are formed through lots of nanofibers, which have higher glass transition temperature and dimensional stability. Microporous membranes fabricated by biaxial stretching have excellent mechanical strength, and the processed clothing fabrics are flat and upright [60], exhibiting excellent aesthetics. However, due to their high tensile strength, they are difficult to deform, resulting in the poor elasticity of clothing. Additionally, this method not only has a complicated machining process and high cost, but is also not conducive to adjusting pore size well, seriously limiting the development of WBMs.

### 4.3. Electrospinning

Another method of fabricating WBMs is electrospinning, the apparatus for which is depicted in Figure 5d [54]. Process parameters involved in electrospinning mainly include concentration, viscosity, conductivity, voltage, diameter of spinneret hole, humidity, temperature, etc. [61,62,63]. Electrospinning fibers have a variety of morphological structures, such as multichannel fibers, fibers with Janus, etc., and are widely used in different fields (Figure 5e [56]). Solution properties have great influence on the fiber morphologies and pore structures of electrospinning membranes, which are the key factors affecting its waterproof and breathable properties. By introducing metal salt to increase the conductivity of the spinning solution, finer fibers can be produced. The porous structures of the membranes can be controlled by adjusting the fiber diameter and packing density [64].

The equipment required by electrospinning technology is simple and low-cost, has good compatibility with materials and can directly produce nanoscale fibers. Furthermore, it is the most commonly used and effective method for obtaining WBMs in recent years. The prepared fibers have the advantages of small fiber diameter, large specific surface area and good structural adjustability [65,66]. Electrospinning membranes have abundant micron-scale pore structures, which allow water vapor to pass through and prevent external water from entering, achieving a waterproof and breathable effect. However, due to the instability of the jet during the electrospinning process, the fibers formed are randomly arranged [67], which leads to poor mechanical properties of electrospinning membranes. Generally, the mechanical properties of electrospinning membranes can be improved by adding a filler with good mechanical properties or post-treatment.

## 5. Treatment for Enhancing Water Resistance and Water Vapor Permeability

### 5.1. Doping Modification

In addition to the methods of directly preparing WBMs with hydrophilic polymers or hydrophobic polymers mentioned above, the surface hydrophobicity of nanofibers can be improved by doping low surface energy materials [68,69]. Doping modification changes the wettability of the main polymer nanofibers from hydrophilic to hydrophobic [70], thereby obtaining better waterproof breathable properties. Low surface energy materials are usually modified by post-coating or polymer chain modification of the membrane to improve water resistance and water vapor permeability [71]. Common types involve fluorinated polymers and hydrophobic nanoparticles.

#### 5.1.1. Fluorinated Polymers

As a type of low surface energy material, fluorinated polymers can effectively transform hydrophilic materials into hydrophobic ones. By introducing fluorine groups onto the surface to modify the substrate, the surface energy can be reduced, making the material hydrophobic. Direct fluorination [72] is one of the common methods for introducing fluorine groups on textile surfaces in the presence of fluorine gas or plasma. Fluorinated polymers have many interesting properties, including low surface free energy and good resistance to water and oil. Unfortunately, the synthesis process of WBMs fabricated by fluorination is complex, and the fluorine groups also cause environmental pollution [73].

**Figure 6:**
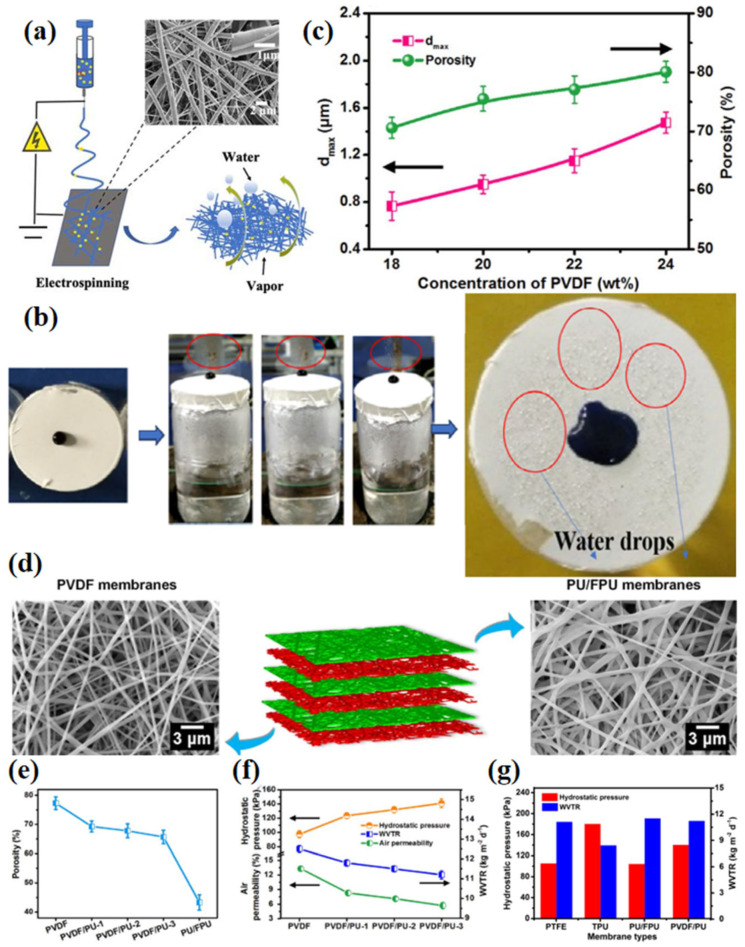
(**a**) Schematic diagram of electrospinning to prepare F-SiO_2_/PU nanofiber membrane [34]; (**b**) experimental demonstration of waterproof and moisture permeability of fiber membrane (It refers to water drops for the red circle.) [34]; (**c**) *d*_max_ and porosity of PVDF fibrous membranes obtained from different solution concentrations [69]; (**d**) illustration of the preparation of composite PVDF/PU fibrous membranes with multilevel porous structure; (**e**) porosity; (**f**) waterproof breathable performance and permeability of PVDF/PU membranes; and (**g**) multilevel porous structured polyvinylidene fluoride/polyurethane fibrous membranes for ultra-high waterproof and breathable applications [69].

For example, Yu et al. [34] developed fluorosilane modified silica (F-SiO_2_) by hydrophobic modification of nanoSiO_2_ with fluorosilane and then added it to synthesized polyurethane to obtain WBM (F-SiO_2_/PU) by electrospinning (Figure 6a,b). The water contact angle of the composite nanofiber membrane increased with an increase in the amount of modified silica. When the amount of F-SiO_2_ was set at 5 wt.%, the water contact angle reached 135°, while the water contact angle of pure PU is only 95°. At the same time, the composite nanofiber membrane had excellent water resistance (hydrostatic pressure of 50 kPa) and water vapor permeability (WVTR of 10.4 kg m^−2^ d^−1^). By constructing a multilevel porous structure, Cui et al. [69] produced an electrospinning fiber membrane with ultra-high waterproof and excellent breathable performance. PVDF concentration regulation produced an optimized fluffy porous structure, good hydrophobicity, moderate hydrostatic pressure of 109 kPa and high WVTR of 12.3 kg m^−2^ d^−1^. In addition, through layer-by-layer combination of polyurethane/fluorinated (PU/FPU) membrane and PVDF membrane, PVDF/PU composite membrane has high hydrostatic pressure (140 kPa) and WVTR (11.3 kg m^−2^ d^−1^), as well as low air permeability of 5.7 mm s^−1^, demonstrating good wind resistance (Figure 6c–f). This excellent waterproof/breathable performance stems from establishment of a new optimization method for WBMs due to the different pore sizes and porosity of the two membranes.

#### 5.1.2. Hydrophobic Nanoparticles

The introduction of hydrophobic nanoparticles is considered a common method for constructing the rough surface structures of non-toxic, harmless and environmentally friendly membranes [74] to improve hydrophobicity. The addition of hydrophobic nanoparticles not only enables nanofibers to have lower surface energy, but also forms a layered roughness on the surface, which is very important for the wetting resistance of the nanofibers. So far, frequently used nanoparticles mainly include organosilicon [75] and silica [76]. The rough structure of the surface of WBMs has a great positive influence on hydrophobicity. However, direct incorporation of nanoparticles into the electrospinning membranes by blending or coating methods often leads to uneven dispersion on the membranes, which limits the improvement of hydrophobicity [77].

### 5.2. Post-Processing

Although the process of direct preparation of WBMs is simple, there are still some limitations. For example, some materials are insoluble in the precursor, making electrospinning difficult; doping modification has a limited effect on improving waterproof performance, etc. In this case, post-treatment is particularly important. Currently, the commonly used post-treatment methods for WBMs include dip coating, vapor deposition and heat treatment [54], which can control the pore structure and improve the performance of WBMs.

#### 5.2.1. Dip Coating

As a practical coating technology, dip coating immerses nanofiber membranes in a modified solution to form a uniform hydrophobic layer on the surface of nanofibers [78]. Dip-coating technology has the advantages of easy operation, uniform coating and a wide source of raw materials [79], and has been widely used in the manufacture of WBMs, filtration membranes and ion exchange membranes. However, the weak interaction between hydrophobic agent and nanofibers leads to unsatisfactory durability of WBMs.

**Figure 7:**
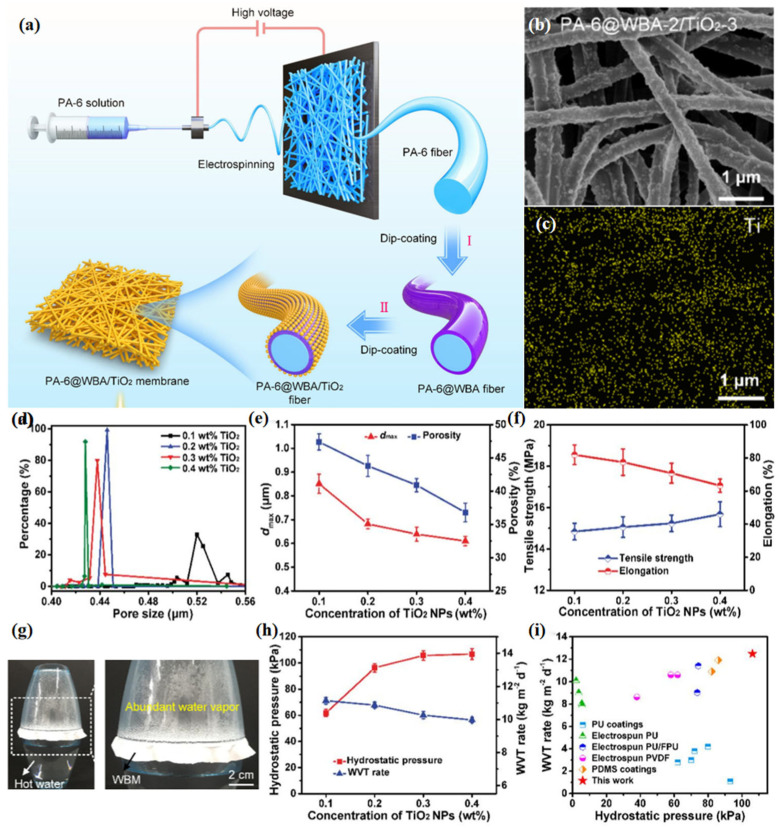
(**a**) Schematic illustration of the preparation procedures of PA-6@WBA/TiO_2_ fluorine-free multifunctional fibrous membranes; (**b**) FE-SEM images of PA-6@WBA-2/TiO_2_-3 membranes; (**c**) elemental mapping image of Ti on the PA-6@WBA-2/TiO_2_-3 membrane; (**d**) pore size distribution; (**e**) *d*_max_ and porosity; (**f**) tensile strength and elongation; (**g**) demonstration of breathable performance; (**h**) hydrostatic pressure and WVTR; and (**i**) comparison of hydrostatic pressure and WVTR of PA-6@WBA-2/TiO_2_ membranes with various TiO_2_ NP concentrations [80].

Using PA-6 fiber membrane as a substrate, Zhao et al. [80] coated fluorine-free water-based alkyl acrylate (WBA) containing long hydrocarbon chains on the fiber membranes with a stepwise dip-coating method to construct a highly hydrophobic surface (Figure 7). Titanium dioxide nanoparticles (TiO_2_ NPs) endow the fiber membranes with the required UV resistance and antibacterial properties, thus successfully fabricating PA-6@WBA/TiO_2_ membranes. With small pore size and high porosity, PA-6@WBA/TiO_2_ membranes exhibited superior performance, with hydrostatic pressure of 106.2 kPa, WVTR of 10.3 kg m^−2^ d^−1^, UPF of 430.5 and bactericidal efficiency > 99.9%. This environmentally friendly WBM provides a valuable reference for the engineering and manufacturing of multifunctional materials.

#### 5.2.2. Vapor Deposition (VD)

The technology of deposition on solid surfaces, called vapor deposition (VD), provides a simple and convenient way for nanofibers to form hydrophobic surfaces without altering the porous structure [81]. VD can be divided into physical vapor deposition (PVD) and chemical vapor deposition (CVD) according to whether chemical reactions occur during deposition [82]. The reaction raw materials required for VD membrane formation are generally relatively easy to obtain, which facilitates controlling the composition and characteristics of the membranes. When the substrate of CVD requires local deposition of a membrane or deposition on a certain surface, CVD is not as convenient as PVD.

**Figure 8:**
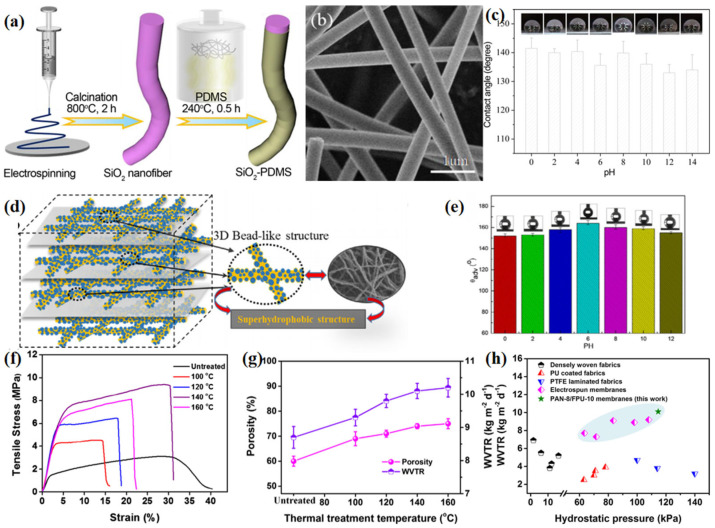
(**a**) Scheme for fabricating hydrophobic SiO_2_–PDMS fibrous membranes [83]; (**b**) SEM images of SiO_2_–PDMS fibers [83]; (**c**) water contact angles with different pH values on SiO_2_–PDMS membrane [83]; (**d**) schematic illustration of the BLNFMs [87]; (**e**) various *θ*_adv_ with different PH solutions [87]; (**f**) stress–strain curves of PAN-8/FPU-10 fibrous membranes fabricated with different thermal treatment temperatures [88]; (**g**) porosity and WVTR of as-prepared PAN-8/FPU-10 fibrous membranes with different thermal treatment temperatures [88]; and (**h**) comparison of hydrostatic pressure and WVTR among conventional waterproof–breathable materials and PAN-8/FPU-10 macro-porous membranes after heated at 140 °C [88].

Recently, Chang et al. [83] reported a hydrophobic SiO_2_–PDMS membrane by VD, which exhibited good oil absorption performance with an oil/water mixture (Figure 8a–c). The PDMS coating had no effect on the porous structure of the fiber membrane but gave the silica nanofibers good hydrophobicity and lipophilicity. At 240 °C, the heated PDMS was converted into steam and deposited onto the surface of silica fibers, forming a hydrophobic layer with a water contact angle of 135°. In addition, SiO_2_–PDMS membranes displayed both good water resistance and water vapor permeability due to their appropriate pore size and hydrophobic surface, making them suitable for use in fields such as filtration membranes, self-cleaning materials and protective clothing. A super-hydrophobic nanofiber membrane with silicon nanoparticles attached was fabricated by Dizge et al. [84], who coated fluoroalkyl silane onto cellulose acetate membrane by CVD. Compared with commercial hydrophobic PVDF membranes, these modified ones exhibit excellent hydrophobic, oleophobic, wettability and stable water vapor flux properties, which could be applied in the field of direct-contact membrane distillation.

#### 5.2.3. Heat Treatment

This is a convenient method to improve the hydrophobicity of WBMs by blending or coating nanoparticles directly onto the electrospinning membrane. However, the uneven dispersion of nanoparticles on the membrane [85], the unstable adhesion between the modifier and the substrate, and the complex preparation process [86] greatly limit their further practical applications. Heat treatment is a simple and effective way to control the structure and properties of WBMs, which can generate a cohesive structure between the fibers, form attractive dense membranes and improve the mechanical properties of WBMs.

When the concentration of silica nanoparticles reached 7.3 wt.%, Liang et al. [87] fabricated a bead-like SiO_2_@PTFE nanofiber membrane showing a super-hydrophobic surface (Figure 8d,e), a water contact angle of 155°, and moderate WVTR (9.7 kg m^−2^ d^−1^) and air permeability (4.8 mm s^−1^). However, the potential toxicity and low elasticity of PVDF and PTFE limit their use in clothing, requiring greater safety, comfort and deformation resistance during wear and washing.

Recently, Sheng et al. [88] produced polyacrylonitrile (PAN)/fluorinated polyurethane (FPU) nanofiber composite membranes with enhanced waterproof and breathable properties through the combination of electrospinning and hot reprocessing (Figure 8f–h). The introduction of FPU enriches the nanofiber membrane with a super-hydrophobic surface, water contact angle of 151°, and optimized pore size and porosity. By thermal reprocessing, the original PAN/FPU composite membrane was given a physically adhesive structure, significantly improving its water resistance, breathability and mechanical properties. Due to these effects, the heat-treated PAN/FPU composite membranes exhibited high hydrostatic pressure (114.6 kPa), WVRT (10.1 kg m^−2^ d^−1^) and good tensile strength (9.4 MPa), so they can be used as functional materials for manufacturing filters, outdoor sports clothes and military combat uniforms.

## 6. Applications of WBMs in the Textile Field

The application of WBMs in the textile field is generally realized by compounding them with fabrics and forming waterproof breathable fabrics through lamination technology [89]. The market value of waterproof breathable fabrics was USD 1.43 billion in 2015 and is expected to grow at a rate of approximately 6% per year to USD 2.3 billion by 2024 [3], making them a promising textile material. There are three types of waterproof breathable fabrics [90]: high-density waterproof fabrics, waterproof-coating finished fabrics and WBM composite fabrics. High-density waterproof fabric is made of absorbent and hydrophilic yarn or microfiber synthetic yarn, resulting in a small aperture to maximize the resistance to wind and rain but poor water penetration; waterproof-coated fabrics have excellent water resistance but poor water vapor permeability [91]. Due to excellent water resistance and water vapor permeability, WBMs are laminated onto the fabric support layer and fixed under high temperature and pressure, displaying superior waterproof and breathable properties. Therefore, WBM composite fabrics have become the mainstream product for waterproof breathable textiles in the current market. Recent research progress on WBMs is summarized in Table 1. Waterproof breathable textiles make functional clothing with both water resistance and water vapor permeability, playing an indispensable role in outdoor sports [92], special protection [93], medical conservation [94] and self-cleaning [95] fields.

### 6.1. Daily Outdoor Sportswear

To protect waterproof breathable membranes from physical damage and mechanical wear, they are usually sandwiched between layers of fabric [45]. However, if punctured, their performance and water resistance will be greatly reduced. Outdoor sportswear and casual wear are relatively common waterproof and breathable materials in daily life, which need to provide a higher level of comfort as well as weather protection, because consumers pay more attention to comfort when wearing such high-performance clothing [92]. It is worth mentioning that a hydrostatic pressure of 9.8 kPa and WVTR of 5 kg m^−2^ d^−1^ are the minimum acceptable levels for sportswear and raincoats; otherwise, wearers may experience heat stress.

The waterproof jacket is one of the most widely used and well-known waterproof breathable fabrics in daily life. The main purpose of these jackets is not only to protect people from water and wind, but also to maintain comfort while wearing, which largely depends on the “breathability” of the outer shell. At present, both hydrophilic non-porous and hydrophobic microporous membranes can be used to fabricate waterproof jackets [100]. Elise et al. [101] designed a climbing waterproof jacket specifically developed for women, which combined hydrophilic PU membranes with a lining through lamination technology and waterproofing the edges. The jacket has waterproof protection while still maximizing airflow through the clothing, ensuring the comfort of wearing. Then, hydrophobic microporous membranes, represented by PTFE membranes (such as Gore-Tex) [102], are constructed to act as a filter to block pollutants. A major breakthrough in WBM fabrics is the development of self-sealing fabrics [103], which can solve the problem of reduced waterproofness and breathable performance caused by internal WBM damage after sewing on coats, tents, etc.

In order to ensure that the cloth has high permeability, waterproofing, anti-pollution and antibacterial properties, Liu et al. [104] proposed a multifunctional fabric with cotton fabric as the raw material, one side with a super-hydrophobic treatment and the other side with a porous membrane coating. The super-hydrophobic layer is coated with SiO_2_/polydimethylsiloxane (PDMS), resulting in a water contact angle of 161° and a sliding angle of less than 5°, effectively preventing the entry of external water. The porous layer is cellulose acetate film with micropores, and the high porosity ensures the breathability of the fabric. Then, a metallic silver network deposited on the porous layer achieves the triple effect of infrared reflection, flexible heating and antibacterial properties. The fabric is likely to be widely used in fields such as clothing and outdoor sports.

### 6.2. Special Protective Clothing

As a waterproof and breathable layer of protective clothing, WBMs also play a great role in the field of protection [105], helping staff who need to work at high temperatures to effectively discharge surface sweat and reduce the occurrence of heat stress [106]. Heart failure caused by heat stress is one of the most common causes of death of fire personnel, so the WVTR of firefighting clothing should be around 10 kg m^−2^ d^−1^ to ensure the safety of high-temperature operation. In military operations and terrorist attacks, soldiers are at high risk of being harmed by toxic substances [93]. To ensure the life safety of workers in dangerous environments, there are high requirements for the anti-liquid permeability of fabrics, which can be achieved by improving the waterproof and corrosion resistance of WBMs. As protective clothing, it is necessary to have good elasticity and wear resistance to prevent puncturing with sharp objects from the outside. Membranes based on inorganic chalcogenide nanotubes and metal oxide nanoparticles [3] are excellent candidates for bulletproof vests.

**Figure 9:**
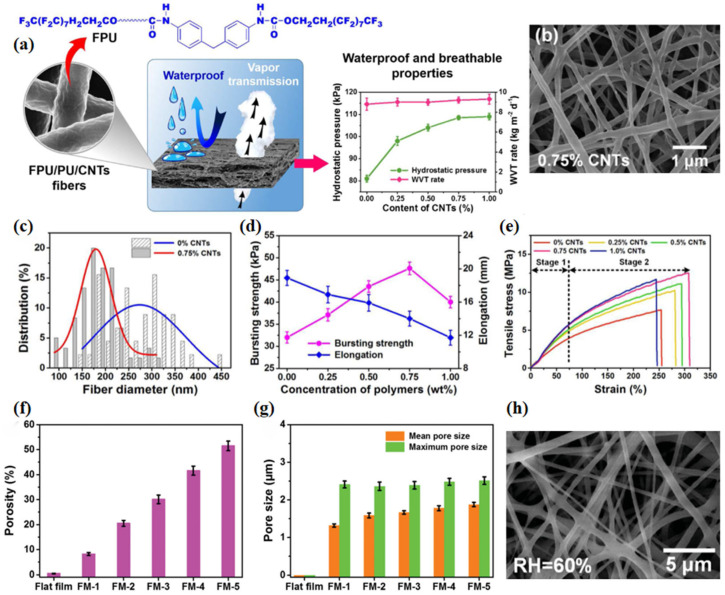
(**a**) Waterproof and breathable properties of FPU/PU fibrous membranes [96]; (**b**) FE-SEM images of FPU/PU/CNTs—0.75% fibrous membranes [96]; (**c**) fiber diameter distribution of FPU/PU/CNT fibrous membranes containing 0% and 0.75% CNTs [96]; (**d**) bursting strength and elongation; (**e**) tensile stress−strain curves of the FPU/PU/CNT fibrous membranes containing different contents of CNT [96]; (**f**) porosity; (**g**) pore size of the PU/FPU flat film and fibrous membranes [98]; (**h**) FE-SEM images of the 60% RH PU/FPU fibrous membranes [98].

Li et al. [96] proposed an easy method to produce a novel electrospinning composite fiber membrane with high waterproof and breathable properties, which is composed of polyurethane (PU), end-fluorinated polyurethane (FPU) and carbon nanotubes (CNT) (Figure 9a–e). Based on the use of FPU and CNT, more rough structures were formed on the fiber surface, resulting in a hydrostatic pressure of up to 108 kPa and a WVRT exceeding 9.2 kg m^−2^ d^−1^, with good bursting strength (47.6 kPa) and tensile strength (12.5 MPa). The results showed that the FPU/PU/CNT fiber membrane was a promising candidate material for protective clothing.

Furthermore, using PU and FPU as raw materials, Li et al. [98] obtained a hydrophobic fiber membrane with breathable and waterproof properties through electrospinning (Figure 9f–h). The porous structure of the membrane was adjusted by modulating relative humidity (RH) and electrospinning time. When the RH reached 60%, the membrane showed 60% porosity and 1.2 μm pore size. The composite fiber membrane was waterproof and breathable, and had good mechanical properties (tensile strength: 10 MPa; elongation: 353%), so it could be used as a new material for the manufacture of protective clothing.

### 6.3. Wound Dressing

For occupations that must resist fluid penetration, such as medical personnel and firefighters, protective clothing requires a higher degree of waterproofing [94]. Because of the long-term use of surgical gowns, it is important to ensure the safety and comfort of doctors during an operation [107]. Therefore, WBMs play a crucial role not only in preventing a patient’s blood from infecting the doctor but also in maintaining the doctor’s comfort and dryness during surgery. Excellent WVTR is also important for wound healing [108] as it controls the moisture content of the wound to promote the proliferation of epidermal cells and fibroblasts. Therefore, one way to create the ideal microenvironment around a wound is to apply WBMs as a wound dressing. In addition, patients often need to recover in bed for a long time after receiving treatment. Traditional hospital sheets and hospital gowns are made of cotton fabric, but cotton fabric generally has good hydrophilicity and poor sweat release [97], which can easily cause bacteria and viruses to multiply on the sheets and hospital gowns, which is also not conducive to the postoperative recovery of patients. Consequently, the development of medical supplies with good protection and water vapor permeability to protect the common health of medical workers and patients is an urgent demand in the medical health field.

**Figure 10:**
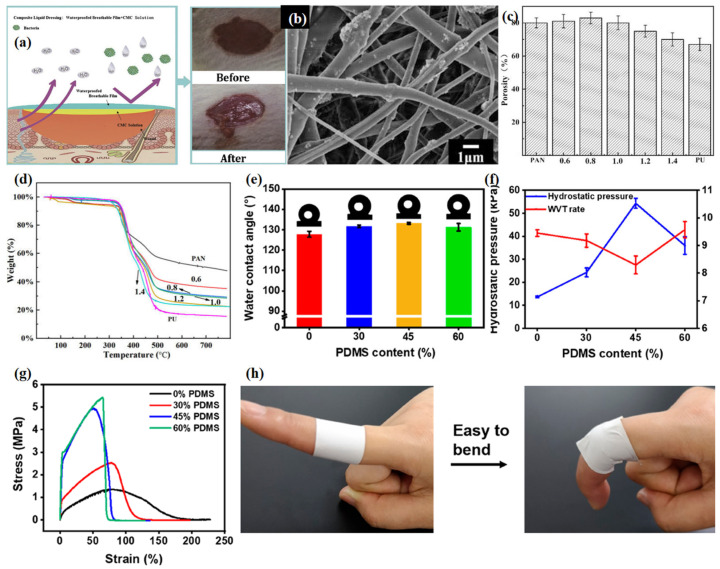
(**a**) A double-layered wound dressing composed of a CMC membrane and PVB WPBL on top of it [108]; (**b**) SEM images of (PU/F–SiO_2_)/PAN nanofiber membranes; (**c**) porosity; (**d**) TGA curves of PAN, PU nanofiber membranes and (PU/F–SiO_2_)/PAN nanofiber membranes with different feeding speed ratios [109]; (**e**) water contact angles; (**f**) hydrostatic pressure and WVTR; (**g**) stress−strain curves of PVB/PDMS fibrous membranes with different PDMSs [94]; (**h**) photographs of PVB/PDMS—45% fibrous membranes applied on a finger knuckle [94].

Yu et al. [109] mixed fluorosilane-modified silica nanoparticles (F-SiO_2_) with synthetic polyurethane (PU) solution, then compounded it with polyacrylonitrile (PAN) solution, and obtained a new hydrophobic microporous nanofiber membrane by electrospinning (Figure 10b–d). The obtained PAN/(F-SiO_2_/PU) nanofiber microporous membrane achieved strong tensile strength (19.5 MPa), good WVRT (10.3 kg m^−2^ d^−1^), good water contact angle (137.2°), and excellent thermal stability and mechanical properties. It is believed that the enhanced PAN/(F-SiO_2_/PU) nanofiber composite membranes have potential application prospects in medical products such as chemical protective clothing, military combat clothing and self-cleaning materials.

In view of the green manufacturing method, Guo et al. [94] developed a skin-like waterproof breathable polyvinyl butyral (PVB) embedded polydimethylsiloxane (PDMS) fiber membrane (Figure 10e–h). The addition of hydrophobic agent PDMS enhanced the surface hydrophobicity of the fiber membrane, with a maximum hydrostatic pressure of 54.32 kPa, a WVRT of 8.98 kg m^−2^ d^−1^ and an increase in mechanical strength of 4.95 MPa. The developed fibrous membrane provided functions similar to human skin and allowed for adequate stretching at the joint location, enabling better design of waterproof, breathable and stretchable wound dressings. Of course, there are also many studies on cellulose membranes in the field of wound healing. Shi et al. [110] synthesized a cross-linked cellulose membrane (CEM) constructed with epichlorohydrin (EP) crosslinking agent, which has high fracture strength (137.4MPa), adaptive permeability and excellent biocompatibility. Compared with the original cellulose membrane, the light transmittance and water repellency of the crosslinked membrane have been improved to some extent.

### 6.4. Bionic Textiles

**Figure 11:**
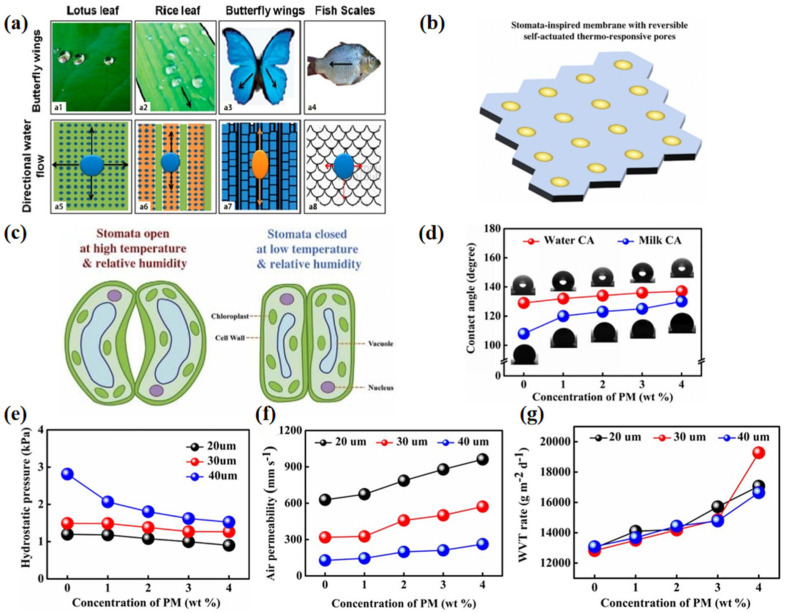
(**a**) Schematic illustration of water flow on natural super-hydrophobic surfaces [47]; (**b**) scheme showing a stomata-inspired membrane with reversible self-actuated thermo-responsive pores; (**c**) the respirational pore structure of a leaf (stomata) that can open and close in response to light, temperature and humidity for gas exchange [45]; (**d**) WCAs and MCAs; (**e**) hydrostatic pressure; (**f**) air permeability; and (**g**) WVTRs of pristine TPU and TPU-PMx NFMs [115].

In recent years, bionic intelligent textiles have gradually entered the vision of people with the development of bionics [111]. Because the damage caused by washing to a fabric’s waterproof and breathable properties is permanent, self-cleaning waterproof breathable textiles have become a popular research direction [112]. Researchers have noticed that the waxy surface structure of lotus leaves has a low adhesion to water droplets, thus possessing good waterproofing and self-cleaning capabilities, which is known as the “lotus effect” [99,113]. In addition, the wings and scales of many animals, as well as the leaves of plants, exhibit cleaning properties (Figure 11a [47]). By controlling the assembly of carbon nanotubes, silicon and polymer [114], the special rough structure of the lotus surface is imitated, so that the water contact angle is greater than 150° and the purpose of self-cleaning is achieved. Figure 11b,c [45] demonstrate the stomatal structure of simulated leaves, as well as the WBMs that can automatically adjust water vapor permeability by closing or opening their stomata according to the prepared environment.

Introducing photochromic microcapsules (PM) into an electrospinning thermoplastic polyurethane (TPU) membrane, a new waterproof and breathable membrane with good photochromic properties was prepared by Liu et al. [115] (Figure 11d–g). Compared with the original TPU samples, the composite TPU/PM membrane had reversible photochromic properties. In addition, the composite membrane not only had a water contact angle of 137° and milk contact angle of 130°, but also had moderate comprehensive properties: for instance, WVRT of 19.3 kg m^−2^ d^−1^, high air permeability of 962 mm s^−1^, low water resistance of 2.813 kPa and tensile strength of 12.08 MPa. The convenience and efficiency of the manufacturing process will allow the mass production of multifunctional fibrous membranes, which can also be applied to photochromic skin, “chameleon” tablecloths and protective clothing, among other materials. Furthermore, Liu et al. [104] created a wearable multifunctional textile by combining antibacterial material with waterproof, breathable membranes. In consequence, the combination of waterproof, breathable, photochromic, antibacterial, heat insulation, electronics and other aspects provides new inspiration for the structural design of intelligent textiles, which will also be the development trend of the textile industry in the future.

## 7. Conclusions and Perspectives

In general, improvement of the comprehensive performance of WBMs has made the development of large-scale production technology possible. WBMs are usually classified as hydrophilic non-porous membranes and hydrophobic microporous membranes, but due to the lack of pore structure in hydrophilic non-porous membranes, hydrophobic microporous membranes are more widely applied. Compared with traditional pure WBMs (such as PU membranes), composite membranes have developed rapidly in recent years, possessing multi-functional modification and environment-friendly characteristics. In addition to obtaining composite WBMs with better performance by combining different polymers, the waterproof and breathable properties of WBMs can also be enhanced through doping fluorinated polymers or inorganic nanoparticles and post-treatment methods. At present, WBMs are widely used in many waterproof and breathable scenes, such as fire protection, aviation, food packaging, seawater desalination, gas–liquid separation and especially the textile field as described in this paper. Although the research results achieved by WBMs cannot be ignored, many issues related to energy and health still need to be solved. For example, the commonly used lamination technology combining WBMs with textile materials still has many defects, such as the inability to control the durability of the material, wearing comfort, etc. At the same time, WBMs prepared by electrospinning have poor mechanical properties, are easy to break, or wear, which seriously affects their service life. Moreover, most WBMs are prepared with organic solvents, such as N, N-dimethylformamide (DMF), tetrahydrofuran (THF), etc., causing serious environmental pollution. In short, although there are still many challenges, the development of WBMs in the textile field is still exciting and demonstrates great potential. If issues related to health and energy can be solved, this may make our lives more convenient.

## Figures and Tables

**Table 1:** Summary of WCA, hydrostatic pressure and WVRT of WBMs.

Materials	WCA (°)	Hydrostatic Pressure (kPa)	WVRT (kg m^−2^ d^−1^)	Refs.
F-SiO_2_/PU	135	50	10.4	[34]
PVDF/PU	/	140	11.3	[68]
PVDF	148	109	12.3	[68]
PA-6@WBA/TiO_2_	129.8	106.2	10.3	[79]
SiO_2_@PTFE	155	/	9.7	[86]
PAN/FPU	151	114.6	10.1	[87]
FPU/PU/CNTs	/	108	9.2	[93]
PU/FPU	117	86	11.9	[96]
(PU/F–SiO_2_)/PAN	137.2	/	10.3	[97]
PVB/PDMS	133.16	54.32	8.98	[98]
TPU/PM	137	2.81	19.3	[99]
PU/SSNPs/TEOS	138	23.5	5.91	[89]

## Data Availability

Not applicable.

## References

[1] Scott C.L., Henri S., Guilliams M. (2014). Mononuclear phagocytes of the intestine, the skin, and the lung. Immunol. Rev..

[2] Zhong W., Xing M.M.Q., Pan N., Maibach H.I. (2006). Textiles and human skin, microclimate, cutaneous reactions: An overview. Cutan. Ocul. Toxicol..

[3] Gugliuzza A., Drioli E. (2013). A review on membrane engineering for innovation in wearable fabrics and protective textiles. J. Membr. Sci..

[4] Ferreira A., Novoa P.R.O., Marques A.T. (2016). Multifunctional Material Systems: A state-of-the-art review. Compos. Struct..

[5] Ahn H.W., Park C.H., Chung S.E. (2011). Waterproof and breathable properties of nanoweb applied clothing. Text. Res. J..

[6] Zhang S.C., Liu H., Yu J.Y., Luo W.J., Ding B. (2016). Microwave structured polyamide-6 nanofiber/net membrane with embedded poly(m-phenylene isophthalamide) staple fibers for effective ultrafine particle filtration. J. Mater. Chem. A.

[7] Gorji M., Jeddi A.A.A., Gharehaghaji A.A. (2012). Fabrication and characterization of polyurethane electrospun nanofiber membranes for protective clothing applications. J. Appl. Polym. Sci..

[8] Huizing R., Mérida W., Ko F. (2014). Impregnated electrospun nanofibrous membranes for water vapour transport applications. J. Membr. Sci..

[9] Fu Z., Li K., Pu L., Ge B., Chen Z. (2016). Waterproof Breathable Membrane Used as Gas Diffusion Layer in Activated Carbon Air Cathode Microbial Fuel Cells. Fuel Cells.

[10] Eynolghasi M.B., Mohammadi T., Tofighy M.A. (2022). Fabrication of polystyrene (PS)/cyclohexanol-based carbon nanotubes (CNTs) mixed matrix membranes for vacuum membrane distillation application. J. Environ. Chem. Eng..

[11] Shi S., Si Y., Han Y., Wu T., Iqbal M.I., Fei B., Li R.K.Y., Hu J., Qu J. (2022). Recent Progress in Protective Membranes Fabricated via Electrospinning: Advanced Materials, Biomimetic Structures, and Functional Applications. Adv. Mater..

[12] Jiang Y., Liang P., Huang X., Ren Z.J. (2018). A novel microbial fuel cell sensor with a gas diffusion biocathode sensing element for water and air quality monitoring. Chemosphere.

[13] Yu Q., Xiong R., Li C., Pecht M.G. (2019). Water-Resistant Smartphone Technologies. IEEE Access.

[14] Wang L., Cao T., Dykstra J.E., Porada S., Biesheuvel P.M., Elimelech M. (2021). Salt and Water Transport in Reverse Osmosis Membranes: Beyond the Solution-Diffusion Model. Environ. Sci. Technol..

[15] Wang L., He J., Heiranian M., Fan H., Song L., Li Y., Elimelech M. (2023). Water transport in reverse osmosis membranes is governed by pore flow, not a solution-diffusion mechanism. Sci. Adv..

[16] Al-Furaiji M., Kadhom M., Kalash K., Waisi B., Albayati N. (2020). Preparation of thin-film composite membranes supported with electrospun nanofibers for desalination by forward osmosis. Drink. Water Eng. Sci..

[17] Alkhudhiri A., Darwish N., Hilal N. (2012). Membrane distillation: A comprehensive review. Desalination.

[18] Xu M.D., Cheng J.X., Du X.F., Guo Q., Huang Y., Huang Q.L. (2022). Amphiphobic electrospun PTFE nanofibrous membranes for robust membrane distillation process. J. Membr. Sci..

[19] Ye H., Wang J., Wang Y., Chen X.-P., Shi S.-P. (2013). Effects of simultaneous chemical cross-linking and physical filling on separation performances of PU membranes. Iran. Polym. J..

[20] Sheng J., Zhang M., Xu Y., Yu J., Ding B. (2016). Tailoring Water-Resistant and Breathable Performance of Polyacrylonitrile Nanofibrous Membranes Modified by Polydimethylsiloxane. ACS Appl. Mater. Interfaces.

[21] Bagherzadeh R., Latifi M., Najar S.S., Tehran M.A., Gorji M., Kong L. (2012). Transport properties of multi-layer fabric based on electrospun nanofiber mats as a breathable barrier textile material. Text. Res. J..

[22] Xiang H., Wang B., Zhong M., Liu W., Yu D., Wang Y., Tam K.C., Zhou G., Zhang Z. (2022). Sustainable and Versatile Superhydrophobic Cellulose Nanocrystals. ACS Sustain. Chem. Eng..

[23] Rouhani S.T., Fashandi H. (2018). Breathable dual-layer textile composed of cellulose dense membrane and plasma-treated fabric with enhanced comfort. Cellulose.

[24] Pang H., Tian K., Li Y., Su C., Duan F., Xu Y. (2021). Super-hydrophobic PTFE hollow fiber membrane fabricated by electrospinning of Pullulan/PTFE emulsion for membrane deamination. Sep. Purif. Technol..

[25] Wu J., Wang N., Wang L., Dong H., Zhao Y., Jiang L. (2012). Electrospun Porous Structure Fibrous Film with High Oil Adsorption Capacity. ACS Appl. Mater. Interfaces.

[26] Gu J., Xu S., Lu X., Ma R., Zhang S., Zheng S., Wang H., Shen H. (2023). Study on the membrane formation mechanism of PVDF/PVDF-CTFE blends. J. Taiwan Inst. Chem. Eng..

[27] Serbezeanu D., Popa A.M., Stelzig T., Sava I., Rossi R.M., Fortunato G. (2015). Preparation and characterization of thermally stable polyimide membranes by electrospinning for protective clothing applications. Text. Res. J..

[28] Eykens L., De Sitter K., Dotremont C., Pinoy L., Van der Bruggen B. (2016). Characterization and performance evaluation of commercially available hydrophobic membranes for direct contact membrane distillation. Desalination.

[29] Manshahia M., Das A. (2014). Moisture management of high active sportswear. Fibers Polym..

[30] Akhtar F.H., Kumar M., Vovusha H., Shevate R., Villalobos L.F., Schwingenschlögl U., Peinemann K.-V. (2019). Scalable Synthesis of Amphiphilic Copolymers for CO_2_- and Water-Selective Membranes: Effect of Copolymer Composition and Chain Length. Macromolecules.

[31] Pantelic J., Teitelbaum E., Bozlar M., Kim S., Meggers F. (2018). Development of moisture absorber based on hydrophilic nonporous membrane mass exchanger and alkoxylated siloxane liquid desiccant. Energy Build..

[32] Zhu F.-L., Zhou Y., He J.-X. (2013). Moisture transport through non-porous hydrophilic membranes used in protective clothing. Therm. Sci..

[33] Mukhopadhyay A., Midha V.K. (2008). A Review on Designing the Waterproof Breathable Fabrics Part I: Fundamental Principles and Designing Aspects of Breathable Fabrics. J. Ind. Text..

[34] Yu Y.G., Liu Y., Zhang F.L., Jin S.X., Xiao Y.Q., Xin B.J., Zheng Y.S. (2020). Preparation of Waterproof and Breathable Polyurethane Fiber Membrane Modified by Fluorosilane-modified Silica. Fibers Polym..

[35] Marathe R., Tatiya P., Chaudhari A., Lee J., Mahulikar P., Sohn D., Gite V. (2015). Neem acetylated polyester polyol—Renewable source based smart PU coatings containing quinoline (corrosion inhibitor) encapsulated polyurea microcapsules for enhance anticorrosive property. Ind. Crop. Prod..

[36] De Smet D., Wéry M., Uyttendaele W., Vanneste M. (2021). Bio-Based Waterborne PU for Durable Textile Coatings. Polymers.

[37] Vincent O., Marguet B., Stroock A.D. (2017). Imbibition Triggered by Capillary Condensation in Nanopores. Langmuir.

[38] Ge J., Si Y., Fu F., Wang J., Yang J., Cui L., Ding B., Yu J., Sun G. (2013). Amphiphobic fluorinated polyurethane composite microfibrous membranes with robust waterproof and breathable performances. RSC Adv..

[39] Gong X., Yin X., Wang F., Liu X., Yu J., Zhang S., Ding B. (2023). Electrospun Nanofibrous Membranes: A Versatile Medium for Waterproof and Breathable Application. Small.

[40] Li Y., Zhang C., Zhu L., Ahmad Z., Li J., Chang M. (2019). Elastic antibacterial membranes comprising particulate laden fibers for wound healing applications. J. Appl. Polym. Sci..

[41] Su Y., Li R., Yang J., Xiang C., Song G., Li J. (2019). Influence of Transport Properties of Laminated Membrane-fabric on Thermal Protective Performance Against Steam Hazard. Fibers Polym..

[42] Natarajan E., Santhosh M.S., Markandan K., Sasikumar R., Saravanakumar N., Dilip A.A. (2022). Mechanical and wear behaviour of PEEK, PTFE and PU: Review and experimental study. J. Polym. Eng..

[43] Lakshmi R., Bharathidasan T., Bera P., Basu B.J. (2012). Fabrication of superhydrophobic and oleophobic sol–gel nanocomposite coating. Surf. Coat. Technol..

[44] Lü X., Wang X., Guo L., Zhang Q., Guo X., Li L. (2016). Preparation of PU modified PVDF antifouling membrane and its hydrophilic performance. J. Membr. Sci..

[45] Tehrani-Bagha A.R. (2019). Waterproof breathable layers—A review. Adv. Colloid Interface Sci..

[46] Jiang Q., Wang Y., Xie Y., Zhou M., Gu Q., Zhong Z., Xing W. (2022). Silicon carbide microfiltration membranes for oil-water separation: Pore structure-dependent wettability matters. Water Res..

[47] Ahmad I., Kan C.-W. (2016). A Review on Development and Applications of Bio-Inspired Superhydrophobic Textiles. Materials.

[48] Ellinas K., Dimitrakellis P., Sarkiris P., Gogolides E. (2021). A Review of Fabrication Methods, Properties and Applications of Superhydrophobic Metals. Processes.

[49] Gao S.J., Shi Z., Bin Zhang W., Zhang F., Jin J. (2014). Photoinduced Superwetting Single-Walled Carbon Nanotube/TiO_2_ Ultrathin Network Films for Ultrafast Separation of Oil-in-Water Emulsions. ACS Nano.

[50] Si Y., Fu Q., Wang X., Zhu J., Yu J., Sun G., Ding B. (2015). Superelastic and Superhydrophobic Nanofiber-Assembled Cellular Aerogels for Effective Separation of Oil/Water Emulsions. ACS Nano.

[51] Lewandowski A., Wilczynski K. (2018). General model of polymer melting in extrusion process. Polimery.

[52] Repka A.M., Shah S., Lu J., Maddineni S., Morott J., Patwardhan K., Mohammed N.N. (2012). Melt extrusion: Process to product. Expert Opin. Drug Deliv..

[53] Saerens L., Vervaet C., Remon J.P., De Beer T. (2014). Process monitoring and visualization solutions for hot-melt extrusion: A review. J. Pharm. Pharmacol..

[54] Yu X., Wu X., Si Y., Wang X., Yu J., Ding B. (2019). Waterproof and Breathable Electrospun Nanofibrous Membranes. Macromol. Rapid Commun..

[55] Gugliuzza A., Fabiano R., Garavaglia M., Spisso A., Drioli E. (2006). Study of the surface character as responsible for controlling interfacial forces at membrane–feed interface. J. Colloid Interface Sci..

[56] Yang X., Wang J., Guo H., Liu L., Xu W., Duan G. (2020). Structural design toward functional materials by electrospinning: A review. E-Polymers.

[57] Chen X., Xiang D., Zhou Z., Wu Y., Li H., Zhao C., Li Y. (2021). Biaxial Stretching of Polymer Nanocomposites: A Mini-Review. Front. Mater..

[58] Gontarek-Castro E., Castro-Muñoz R., Lieder M. (2022). New insights of nanomaterials usage toward superhydrophobic membranes for water desalination via membrane distillation: A review. Crit. Rev. Environ. Sci. Technol..

[59] Ouchiar S., Stoclet G., Cabaret C., Addad A., Gloaguen V. (2016). Effect of biaxial stretching on thermomechanical properties of polylactide based nanocomposites. Polymer.

[60] Meng L.-P., Lin Y.-F., Xu J.-L., Chen X.-W., Li X.-Y., Zhang Q.-L., Zhang R., Tian N., Li L.-B. (2015). A Universal equipment for biaxial stretching of polymer films. Chin. J. Polym. Sci..

[61] Sas I., Gorga R.E., Joines J.A., Thoney K.A. (2012). Literature review on superhydrophobic self-cleaning surfaces produced by electrospinning. J. Polym. Sci. Part B Polym. Phys..

[62] Ismail N., Maksoud F.J., Ghaddar N., Ghali K., Tehrani-Bagha A. (2016). Simplified modeling of the electrospinning process from the stable jet region to the unstable region for predicting the final nanofiber diameter. J. Appl. Polym. Sci..

[63] Maksoud F.J., Lameh M., Fayyad S., Ismail N., Tehrani-Bagha A.R., Ghaddar N., Ghali K. (2018). Electrospun waterproof breathable membrane with a high level of aerosol filtration. J. Appl. Polym. Sci..

[64] Liao X., Dulle M., Silva J.M.D.S.E., Wehrspohn R.B., Agarwal S., Förster S., Hou H., Smith P., Greiner A. (2019). High strength in combination with high toughness in robust and sustainable polymeric materials. Science.

[65] Tebyetekerwa M., Xu Z., Yang S., Ramakrishna S. (2020). Electrospun Nanofibers-Based Face Masks. Adv. Fiber Mater..

[66] Liu H.-Y., Xu L., Si N., Tang X.-P. (2014). Thermal treatment for nanofibrous membrane. Therm. Sci..

[67] Li J., Liu X., Sun H., Wang L., Zhang J., Deng L., Ma T. (2020). An Optical Fiber Sensor Coated with Electrospinning Polyvinyl Alcohol/Carbon Nanotubes Composite Film. Sensors.

[68] De Bruycker K., Delahaye M., Cools P., Winne J., Du Prez F.E. (2017). Covalent Fluorination Strategies for the Surface Modification of Polydienes. Macromol. Rapid Commun..

[69] Cui H.M., Li Y.Y., Zhao X.L., Yin X., Yu J.Y., Ding B. (2017). Multilevel porous structured polyvinylidene fluoride/polyurethane fibrous membranes for ultrahigh waterproof and breathable application. Compos. Commun..

[70] Mao X., Chen Y., Si Y., Li Y., Wan H., Yu J., Sun G., Ding B. (2013). Novel fluorinated polyurethane decorated electrospun silica nanofibrous membranes exhibiting robust waterproof and breathable performances. RSC Adv..

[71] Peng C., Chen Z., Tiwari M.K. (2018). All-organic superhydrophobic coatings with mechanochemical robustness and liquid impalement resistance. Nat. Mater..

[72] Navarrini W., Venturini F., Tortelli V., Basak S., Pimparkar K.P., Adamo A., Jensen K.F. (2012). Direct fluorination of carbon monoxide in microreactors. J. Fluor. Chem..

[73] Arvaniti O.S., Hwang Y., Andersen H.R., Stasinakis A.S., Thomaidis N.S., Aloupi M. (2015). Reductive degradation of perfluorinated compounds in water using Mg-aminoclay coated nanoscale zero valent iron. Chem. Eng. J..

[74] Gao J., Huang X., Xue H., Tang L., Li R.K. (2017). Facile preparation of hybrid microspheres for super-hydrophobic coating and oil-water separation. Chem. Eng. J..

[75] Hammami M.A., Croissant J.G., Francis L., Alsaiari S.K., Anjum D.H., Ghaffour N., Khashab N.M. (2017). Engineering Hydrophobic Organosilica Nanoparticle-Doped Nanofibers for Enhanced and Fouling Resistant Membrane Distillation. ACS Appl. Mater. Interfaces.

[76] Jin S., Park Y., Park C.H. (2016). Preparation of breathable and superhydrophobic polyurethane electrospun webs with silica nanoparticles. Text. Res. J..

[77] Yu X., Li Y., Wang X., Si Y., Yu J., Ding B. (2020). Thermoconductive, Moisture-Permeable, and Superhydrophobic Nanofibrous Membranes with Interpenetrated Boron Nitride Network for Personal Cooling Fabrics. ACS Appl. Mater. Interfaces.

[78] Jesswein I., Hirth T., Schiestel T. (2017). Continuous dip coating of PVDF hollow fiber membranes with PVA for humidification. J. Membr. Sci..

[79] Yang Y., Fu W., Chen L., Hou C., Chen X., Zhang X. (2021). One-step dip-coating method for preparation of ceramic nanofiber membrane with high permeability and low cost. J. Eur. Ceram. Soc..

[80] Zhao J., Wang X., Xu Y., He P., Si Y., Liu L., Yu J., Ding B. (2020). Multifunctional, Waterproof, and Breathable Nanofibrous Textiles Based on Fluorine-Free, All-Water-Based Coatings. ACS Appl. Mater. Interfaces.

[81] Wang X., Pan Y., Shen C., Liu C., Liu X. (2018). Facile Thermally Impacted Water-Induced Phase Separation Approach for the Fabrication of Skin-Free Thermoplastic Polyurethane Foam and Its Recyclable Counterpart for Oil-Water Separation. Macromol. Rapid Commun..

[82] Dianat G., Movsesian N., Gupta M. (2020). Vapor Deposition of Functional Porous Polymer Membranes. ACS Appl. Polym. Mater..

[83] Chang M.-J., Chai X.-J., Cui W.-N., Liu J. (2018). Facile Fabrication of Electrospun Silica Nanofibrous Membrane with Hydrophobic, Oleophilic and Breathable Performances. Fibers Polym..

[84] Dizge N., Shaulsky E., Karanikola V. (2019). Electrospun cellulose nanofibers for superhydrophobic and oleophobic membranes. J. Membr. Sci..

[85] Ahmed F.E., Lalia B.S., Hashaikeh R. (2015). A review on electrospinning for membrane fabrication: Challenges and applications. Desalination.

[86] Liang Y., Cheng S., Zhao J., Zhang C., Sun S., Zhou N., Qiu Y., Zhang X. (2013). Heat treatment of electrospun Polyvinylidene fluoride fibrous membrane separators for rechargeable lithium-ion batteries. J. Power Sources.

[87] Liang Y., Ju J., Deng N., Zhou X., Yan J., Kang W., Cheng B. (2018). Super-hydrophobic self-cleaning bead-like SiO_2_@PTFE nanofiber membranes for waterproof-breathable applications. Appl. Surf. Sci..

[88] Sheng J., Li Y., Wang X., Si Y., Yu J., Ding B. (2016). Thermal inter-fiber adhesion of the polyacrylonitrile/fluorinated polyurethane nanofibrous membranes with enhanced waterproof-breathable performance. Sep. Purif. Technol..

[89] Lalia B.S., Kochkodan V., Hashaikeh R., Hilal N. (2013). A review on membrane fabrication: Structure, properties and performance relationship. Desalination.

[90] Ghezal I., Moussa A., Ben Marzoug I., El-Achari A., Campagne C., Sakli F. (2020). Development and Surface State Characterization of a Spacer Waterproof Breathable Fabric. Fibers Polym..

[91] Maity S., Chauhan V., Pandit P., Mondal M.I.H. (2022). 12-Waterproof breathable fabrics and suits. Protective Textiles from Natural Resources.

[92] Kim H.-A. (2021). Water Repellency/Proof/Vapor Permeability Characteristics of Coated and Laminated Breathable Fabrics for Outdoor Clothing. Coatings.

[93] Zhao Y., Wang X., Wang D., Li H., Li L., Zhang S., Zhou C., Zheng X., Men Q., Zhong J. (2020). Preparation and Chemical Protective Clothing Application of PVDF Based Sodium Sulfonate Membrane. Membranes.

[94] Guo Y., Zhou W., Wang L., Dong Y., Yu J., Li X., Ding B. (2019). Stretchable PDMS Embedded Fibrous Membranes Based on an Ethanol Solvent System for Waterproof and Breathable Applications. ACS Appl. Bio Mater..

[95] Sun G.F., Wang P., Jiang Y.X., Sun H.C., Liu T., Li G.X., Yu W., Meng C.Z., Guo S.J. (2023). Bioinspired flexible, breathable, waterproof and self-cleaning iontronic tactile sensors for special underwater sensing applications. Nano Energy.

[96] Li Y., Zhu Z., Yu J., Ding B. (2015). Carbon Nanotubes Enhanced Fluorinated Polyurethane Macroporous Membranes for Waterproof and Breathable Application. ACS Appl. Mater. Interfaces.

[97] Zhou W., Gong X., Li Y., Si Y., Zhang S., Yu J., Ding B. (2021). Waterborne electrospinning of fluorine-free stretchable nanofiber membranes with waterproof and breathable capabilities for protective textiles. J. Colloid Interface Sci..

[98] Li Y., Yang F., Yu J., Ding B. (2016). Hydrophobic Fibrous Membranes with Tunable Porous Structure for Equilibrium of Breathable and Waterproof Performance. Adv. Mater. Interfaces.

[99] Xue C.-H., Chen J., Yin W., Jia S.-T., Ma J.-Z. (2012). Superhydrophobic conductive textiles with antibacterial property by coating fibers with silver nanoparticles. Appl. Surf. Sci..

[100] Wei Y.H., Zhang H.Y., Su Z.W., Tao S.Q., Cao X.J., Wan Z.H., Pan W. (2023). Develop an optimal daily washing care for technical jacket by balancing washing efficiency and functional degradation. J. Eng. Fibers Fabr..

[101] Elise H., Kevin K., Kristen M., Jennifer J. (2022). Dragonfly Jacket Waterproof Jacket and Climbing Pant for Female Rock Climbers. Int. Text. Appar. Assoc. Annu. Conf. Proc..

[102] Sadighzadeh A., Valinejad M., Gazmeh A., Rezaiefard B. (2016). Synthesis of polymeric electrospun nanofibers for application in waterproof-breathable fabrics. Polym. Eng. Sci..

[103] Manning K.C., Kotagama P., Burgin T.P., Rykaczewski K. (2020). Breathable, Stimuli-Responsive, and Self-Sealing Chemical Barrier Material Based on Selectively Superabsorbing Polymer. Ind. Eng. Chem. Res..

[104] Liu Q., Huang J., Zhang J., Hong Y., Wan Y., Wang Q., Gong M., Wu Z., Guo C.F. (2018). Thermal, Waterproof, Breathable, and Antibacterial Cloth with a Nanoporous Structure. ACS Appl. Mater. Interfaces.

[105] Baji A., Agarwal K., Oopath S.V. (2020). Emerging Developments in the Use of Electrospun Fibers and Membranes for Protective Clothing Applications. Polymers.

[106] Knížek R., Karhánková D., Bajzík V., Jirsák O. (2019). Lamination of Nanofibre Layers for Clothing Applications. Fibres Text. East. Eur..

[107] Cai L., Xu L., Si Y., Yu J., Ding B. (2022). Autoclavable, Breathable, and Waterproof Membranes Tailored by Ternary Nanofibers for Reusable Medical Protective Applications. ACS Appl. Polym. Mater..

[108] Xia D.-L.M., Chen Y.-P.M., Wang Y.-F.M., Li X.-D.B., Bao N., He H.M., Gu H.-Y. (2016). Fabrication of Waterproof, Breathable Composite Liquid Dressing and Its Application in Diabetic Skin Ulcer Repair. Adv. Ski. Wound Care.

[109] Yu Y., Zhang F., Liu Y., Zheng Y., Xin B., Jiang Z., Peng X., Jin S. (2020). Waterproof and breathable polyacrylonitrile/(polyurethane/fluorinated-silica) composite nanofiber membrane via side-by-side electrospinning. J. Mater. Res..

[110] Shi S., Zhu K., Chen X., Hu J., Zhang L. (2019). Cross-Linked Cellulose Membranes with Robust Mechanical Property, Self-Adaptive Breathability, and Excellent Biocompatibility. ACS Sustain. Chem. Eng..

[111] Wang S., Liu K., Yao X., Jiang L. (2015). Bioinspired Surfaces with Superwettability: New Insight on Theory, Design, and Applications. Chem. Rev..

[112] Mayser M.J., Bohn H.F., Reker M., Barthlott W. (2014). Measuring air layer volumes retained by submerged floating-ferns *Salvinia* and biomimetic superhydrophobic surfaces. Beilstein J. Nanotechnol..

[113] Park Y., Gutierrez M.P., Lee L.P. (2016). Reversible Self-Actuated Thermo-Responsive Pore Membrane. Sci. Rep..

[114] Moghadam S.G., Parsimehr H., Ehsani A. (2021). Multifunctional superhydrophobic surfaces. Adv. Colloid Interface Sci..

[115] Liu M.-N., Yan X., You M.-H., Fu J., Nie G.-D., Yu M., Ning X., Wan Y., Long Y.-Z. (2018). Reversible photochromic nanofibrous membranes with excellent water/windproof and breathable performance. J. Appl. Polym. Sci..

